# Prenatal diagnosis of a novel pathogenic variation in the *ACAN* gene presenting with isolated shortening of fetal long bones in the second trimester of gestation: a case report

**DOI:** 10.1186/s12884-021-03952-w

**Published:** 2021-06-29

**Authors:** Paolo Toscano, Lavinia Di Meglio, Fortunato Lonardo, Letizia Di Meglio, Laura Letizia Mazzarelli, Carmine Sica, Aniello Di Meglio

**Affiliations:** 1 Department of Neuroscience, Reproductive Sciences and Dentistry, School of Medicine and Surgery Federico II of Naples, University of Naples Federico II, Naples, Italy; 2 Diagnostica Ecografica e Prenatale di A. Di Meglio, Via dei Fiorentini n.21, Naples, Italy; 3 Department of Obstetrics and Gynecology, H. Buzzi, University of Milan, Milan, Italy; 4 Department of Medical Genetics, A.O.R.N. “San Pio”, Benevento, Italy

**Keywords:** Prenatal diagnosis, Skeletal dysplasia, *ACAN*, Aggrecan, Case report

## Abstract

**Background:**

Heterozygous mutations of the ACAN gene are a major cause of different evolutive growth defects in the pediatric population, but were never described as a cause of fetal skeletal dysplasia.

**Case presentation:**

A G1 at 21w + 3d came to our institution for the second-trimester ultrasound and a skeletal dysplasia with prevalent involvement of limb’s rhizomelic tracts was suspected. Amniocentesis followed by CGH-array was performed, with normal results. An examination by NGS of some genes associated with skeletal dysplasias showed a novel pathogenic variant of the *ACAN* gene: c.2677delG.

**Conclusion:**

Sequence variations of *ACAN* were never described as a possible cause of fetal skeletal anomalies to date. In this case report, we describe the first prenatal diagnosis of skeletal dysplasia associated with a pathogenic variant of *ACAN*.

## Background

The prenatal finding of fetal short long bones represents a challenging condition for the physician and the patient, due to its association with skeletal dysplasia, chromosomal abnormalities, and genetic syndromes [1–3]. In the postnatal life, heterozygous pathogenic sequence variations of the *ACAN* gene (coding for the proteoglycan aggrecan) were associated with evolutive growth defects, ranging from mild idiopathic short stature to severe skeletal dysplasias. *ACAN* mutations were considered responsible for 1.4% of cases of idiopathic short stature and 13.8% of cases of short stature and advanced bone age in the pediatric population [4–6]. A literature review on PUBMED and EMBASE did not identify reports of *ACAN* mutations in the antenatal period as a cause of fetal short limbs [4–6].

Here we report the first in-utero diagnosis of a novel heterozygous sequence variation in. the *ACAN* gene in a fetus with severe fetal growth restriction with prevalent reduction of the rhizomelic bones biometry.

## Case presentation

**Fig. 1:**
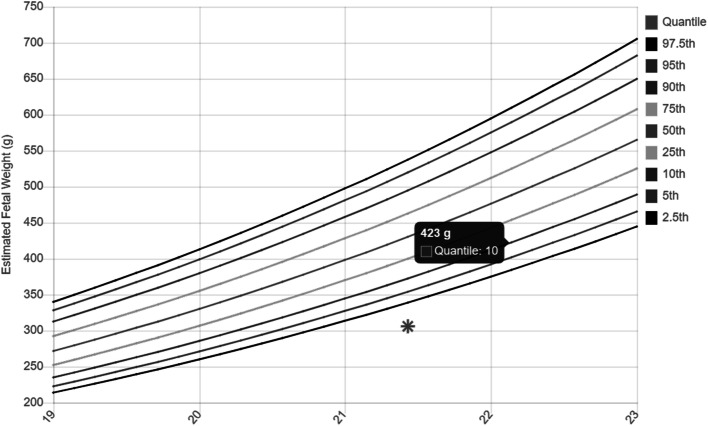
Fetal estimated body weight. Fetal esitimaed body weight under the 5°centile

**Fig. 2:**
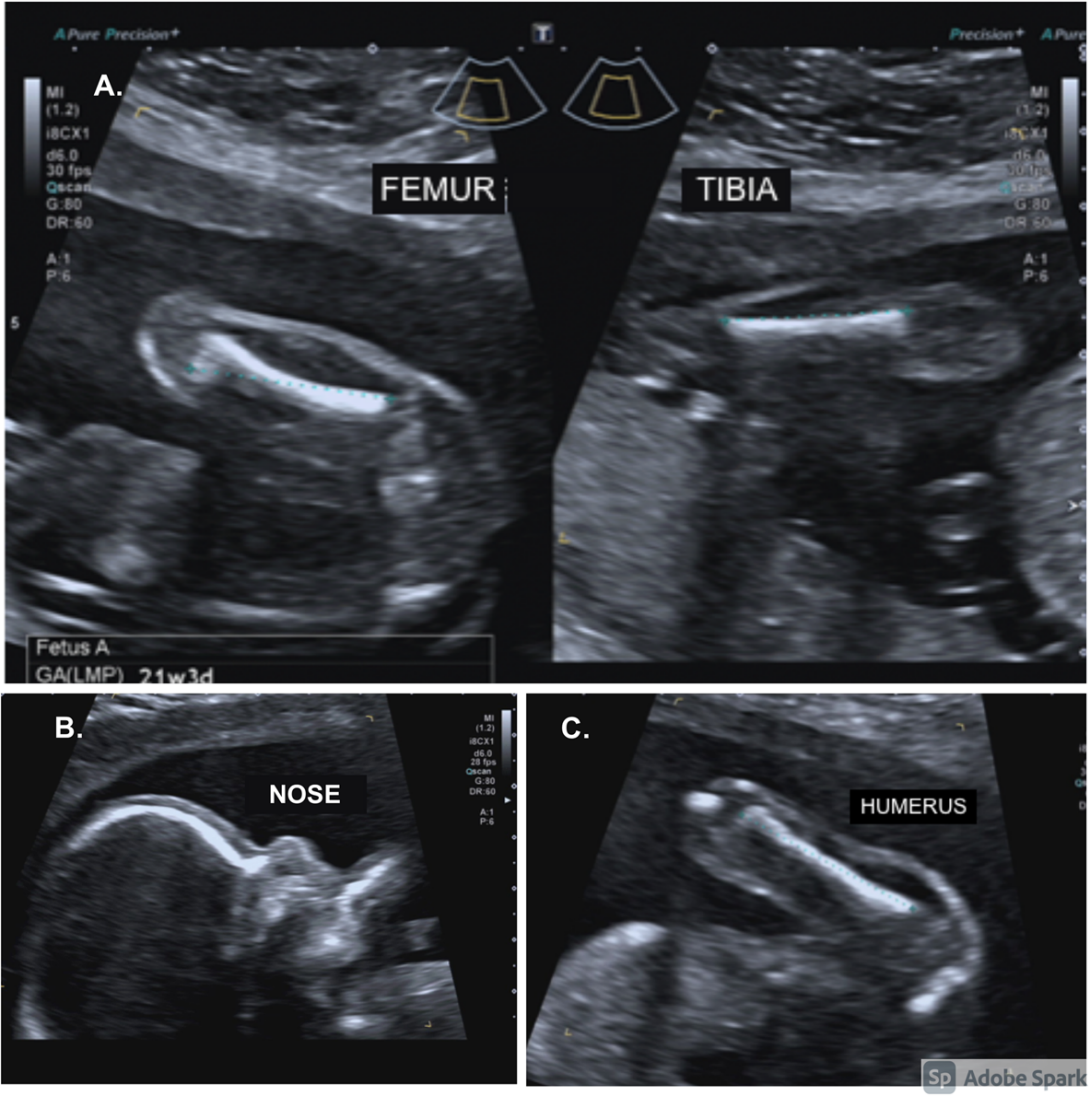
Ultrasounds skeletal findings. **A**. Femur and tibia< 5° centile **B**. Prominence of the forehead with mild nasal hypoplasia. **C**. Homerus 5° centile

A 41-year-old woman (G1) turned to our second-level prenatal ultrasound diagnostic center to undergo the second-trimester screening exam at 21w + 3d of gestation. Besides maternal age > 35 years, in vitro fertilization (IVF) was the only relevant risk factor. Gestational age errors were excluded since this pregnancy was obtained by IVF. Fetal growth was overall ten days smaller than the one attended based on gestational age, with a fetal growth corresponding to 19w + 6d of gestation. In particular, growth’s delay was mostly due to fetal bones: femur was under the third centile, humerus corresponded to the fifth centile and mesomelic bones were all > the 5° centile, with the only exception of the tibia (1.7°centile) (Table 1 and Fig. 1). Fetal anatomy was regular, except for a slight prominence of the forehead with a low nasal bridge and mild nasal hypoplasia, with nasal bone at 17° centile (Fig. 2). No other skeletal anomaly and in particular no polydactyly or bone fractures were detected. The uterine, umbilical, and middle cerebral artery pulsed-wave Doppler measurements were all normal, making less probable placental factor as the cause of growth’s delay. The amniotic fluid was regular. Infective diseases were ruled out based on laboratory tests. Considering the ultrasound findings, a skeletal dysplasia with prevalent involvement of the proximal limbs was suspected.

**Table 1:** Biometry report

GA: 21w + 3d				
	Biometry	Gestational age	Media + − 2DS	Percentile
BPD	44.2 mm	19w	51.7 + −5.0	6.7
HC	183.9 mm	20w + 6d	189.3 + −29.6	42.8
CEREB	22.1 mm	21w + 1d	22.4 + −2	44.0
PV	6.7 mm			
AC	146.0 mm	19w + 2	173.7 + −26	16.1
HL	26.0 mm	19w	32.4 + −4.0	5.5
ULNA	25.3 mm	19w + 3 s	30.9 + − 4.0	8.1
RAD	22.1 mm	19w + 3d	27.9 + −4.0	7.4
FL	27.2 mm	18w + 5d	34.9 + −4.0	2.7
TIB	24.5 mm	19w + 1d	30.9 + −3.0	1.7
FIB	25.1 mm	19w + 5d	29.9 + −3.0	5.5
FOOT	31.0 mm	19w + 2d	37.3 + −4.0	5.8
NB	6.5 mm	19w + 4d	7.2 + −0.7	17.7
EFW	306 g			< 2.5

*GA* gestational age, *BPD* biparietal diameter, *HC* head circumference. *CEREB* cerebellum. *PV* posterior ventricle, *AC* Abdominal circumference, *HL* Homeurs length, *RAD* radial length, *FL* Femur length, *TIB* Tibial length, *FIB* Fibula length, *NB* Nasal bone, *EFW* estimated fetal weight

Amniocentesis was performed to exclude chromosomal, genomic, and genetic anomalies, with particular regard to some genes associated with skeletal dysplasias. Array-based Comparative Genomic Hybridization (CGH-array) and Next Generation Sequencing (NGS) were conducted in parallel. The following databases were used to select the genes that needed to be analyzed: Human Phenotype Ontology, OMIM and/o GeneReviews. CGH-array was performed at the Centro Polidiagnostico s.r.l., Naples, Italy, on a CGH Array platform SurePrint G3 ISCA V2 CGH 8X60K, resolution: 250 kb, Software: Agilent Cytogenomics 5.0.2.5. NGS was performed at Bambino Gesù Pediatric Hospital, Rome, Italy, on a NovaSeq6000 Illumina platform; Genes analyzed: NM_000142.4 (FGFR3) and NM_001135.3 (ACAN). The NGS revealed a heterozygous c.2677delG variant in the *ACAN* gene, which determines the p.Gly893AspfsTer52 protein variation. The mutation is located in exon 12 of the gene and in the first chondroitin sulfate (CS1) attachment domain of the protein. The mutation arose de novo (it wasn’t present in both parent’s DNA). Based on the clinical features associated with *ACAN* mutations a diagnosis of osteochondrodysplasia with short limbs and a possible future involvement of the spine and joints were done. The couple opted for voluntary interruption of pregnancy at 25 weeks of gestational age. No autopsy was performed.

## Discussion

Growth’s delay occurs in almost 10% of pregnancies; the major causes are maternal, placental, fetal, or genetic factors; DNA sequence variations are responsible for almost one-third of growth’s delays [7]. If a growth’s delay is suspected, it is mandatory to collect a complete maternal and familial history, to evaluate previous ultrasound scans, to track fetal growth, to assess gestational age from both the date of the last menstrual period and the first-trimester ultrasound, to evaluate all biometry, to rule out placental problems and infective diseases [7]. In our case report, we describe a fetus that presented 10 days growth delay, mostly due to small fetal rhizomelic bones. Fetal skeletal anomalies occur in 5:1000 pregnancies and, when the gynecologist/fetal radiologist suspects a skeletal anomaly, a genetic consultation should be offered and fetal DNA may be analyzed [3]. In almost 50% of cases, the gene responsible for that anomalies is known, however, in 15% of cases, a variant of uncertain significance is detected, making it difficult for both the geneticist and the gynecologist/fetal radiologist to answer to the couple’s questions [2, 3].

In our case report, amniocentesis followed by CGH-array and NGS was performed and the fetus was found out to be a de novo carrier of a heterozygous pathogenic sequence variation of the *ACAN* gene, which codes for aggrecan. Aggrecan is a proteoglycan component in the extracellular matrix largely present in the articular cartilage and the growth plate [8]. Aggrecan is responsible for the load-bearing capacity of the joints. The glycosaminoglycan side chain attracts counter-ions and water and creates the osmotic swelling pressure that is crucial for biomechanical properties of cartilage under compressive load [8–10]. Moreover, aggrecan is also involved in the autocrine and paracrine regulation of chondrogenesis and osteogenesis [8–10]. Studies conducted on animals with homozygous mutations of the *ACAN* gene showed a premature vascular invasion and ossification of the growth plate with a premature maturation and block of bone elongation [11]. Very interestingly, the tissue in which *ACAN* is expressed at by far the highest levels is the tibial artery (Data Source: GTEx Analysis Release V8, dbGaP Accession phs000424.v8.p2), and in our case, the most compromised bone is the tibia, whose size corresponds to 1.7° centile.

At least 50 pathogenic *ACAN* variations have been identified in patients with highly variable phenotypes of syndromic or non-syndromic short stature (Table 2); no genotype-phenotype relationship was apprised [4–6, 8, 9, 11]. However, in all of these different diseases, the phenotype is characterized by short bones and major involvement of the joints and especially of the spine.

**Table 2:** Diseases associated with ACAN mutations

Isolated Short Stature	
Spondyloepimetaphyseal dysplasia, aggrecan type (OMIM 612813)	
Spondyloepiphyseal dysplasia, Kimberley type (OMIM 608361)	
Short stature and advanced bone age with or without early-onset osteoarthritis and/or osteochondritis dissecans (OMIM 165800)	

Small trials involving GH therapy after birth have been conducted in children with *ACAN* mutations and the therapy resulted in a possible height improvement [6].

In this case, NGS revealed a heterozygous c.2677delG variation in the *ACAN* gene sequence, which resulted in the protein variation p.Gly89eAspfsTer52. The following databases were analyzed to interpret the variant: VarSome, ClinVar, Human Gene mutation database, Leiden Open Variation Database; gnomAD was used to assess the variant’s frequency in the general population (variant not found). The null (frameshift) variant c.2677delG can be classified according to the ACMG guidelines as a Pathogenic variant, since it meets a Very Strong (PVS1), a Moderate (PM2), and a Supporting (PP3) criterion [12]. No mutation involving the *FGFR3* gene was detected.

Despite the variability of the clinical features related to *ACAN* mutations, the genetic variation detected in this case, resulted in a rare form of osteochondrodysplasia with short limbs and possible future involvement of the spine and joints.

From a literature review, *ACAN* mutations have not been reported in the antenatal period, although in the pediatric field they are routinely screened in children with short stature [11].

In this case report, we, therefore, describe a genetic diagnosis never made before in the antenatal period of a pathogenic sequence variation not so far described in the literature**.** More studies involving fetuses with short limbs are advocated to better understand the impact of *ACAN* mutations in the prenatal age.

## Conclusions

Since *ACAN* gene mutations are among the main causes of genetic short stature in the pediatric population, based on our findings, we believe that these should be considered also in the prenatal differential diagnosis of fetal growth’s delay in a fetus with short limbs. The presence of short limbs, even if not all below the pathological value of the 3rd centile, should suggest the possibility of an underlying genetic condition. In case of suspicion of skeletal dysplasia, the *ACAN* gene should be considered. An early diagnosis is particularly important in cases in which, after birth, it is possible to practice without delay a therapy that can positively influence the clinical picture and, ultimately, the quality of life of the patient.

## Data Availability

All data generated or analysed during this study are included in this published article.

## Abbreviations

IVF: in vitro fertilization
CGH-array: Array-based Comparative Genomic Hybridization
NGS: Next Generation Sequencing
